# The Radcliffe Infirmary and Its Chaplain

**Published:** 1900-03-03

**Authors:** 


### The Institutional Workshop.

### THE RADCLIFFE INFIRMARY AND ITS CHAPLAIN.

The discussion anent the chaplain's salary which took place at the last Quarterly Court of Governors of the Radcliffe Infirmary, Oxford, cannot be regarded as altogether satisfactory. It appears that the present chaplain had held office for a period of 44 years, having had a salary of £200 a year, together with the use of a house. It is now proposed that he should retire on a pension of £100 per annum, and that the incoming chaplain should receive the difference, namely, a stipend of £100 a year. In regard to the proposed mode of appointment of the new chaplain, if it really means what it says, we have no special comment to make, the proposal being that the Bishop of Oxford be chaplain to the infirmary, and that he shall appoint an assistant chaplain to perform the duties of the office, which seems reasonable enough. But in regard to the stipend, which is to be £100 per annum, the amount to be reconsidered when the pension ceases to be payable, it may well be asked—as, indeed, it was asked at the meeting—what proportion of the chaplain's time is this £100 per annum to pay for. It was stated at the meeting that the duties of the office would preclude the chaplain from "turning his Sundays to account elsewhere," but that on most other days an hour or two's visiting would be all that would be required. On the answer to this question really hinges the propriety of the proposal as to stipend. If the Bishop had in his pocket some well-to-do and leisurely divine who was willing to undertake the office a stipend of £100 a year might not be too little. It might even be too much. If, on the other hand, the stipend paid by the infirmary were to be used to eke out the meagre salary given to some hard-worked curate who is slaving in his parish the greater portion of his time; or if it were to be given to keep on his feet someone whose main work in life is teaching, then such use of the infirmary stipend could but be described as jobbery; while lastly, if it were the intention of the board to ask for the full services of a properly-qualified chaplain for £100 a year, we cannot but agree with Dr. Collier, who said that he thought that "as it was put this was a scandalous offer to make." No light, however, was thrown upon these points. What seems to us to have been so bad in the management of the whole affair was the total absence of openness and frankness in the manner in which the proposal was placed before the meeting. It was quite evident that a certain number of the governors knew a good deal which was not made public to the rest, and that the votes of these others were given in the dark.

The proposal put forward by the chairman was not the whole proposal as it stood in the minds of those who voted for it. It was hinted, indeed, and then it was stated, that there was some private fund from which the salary would be increased, always, we may suppose, in case the right man should be appointed. Again it was stated by one of the governors that the Bishop had told him "in confidence" the name of the clergyman whom he had in his mind, and he assured the meeting that the choice was a very wise one, and "likely to be acceptable to everybody concerned." But these wonderful secrets, which were of the very essence of the question to be decided, instead of being stated openly to the Board, were treated as confidential, and thus in the end the governors literally bought their pig in a poke.

What is perfectly clear to anyone who reads the published reports of the proceedings at the meeting is that, while ostensibly the appointment of the chaplain was handed over unconditionally to the Bishop, in reality the whole thing had been arranged, and that under the cloak of the perfectly reasonable suggestion that the Bishop of the diocese should have the matter in his own hands, a certain individual was being put into office. This is the sort of thing which, if it came to light in regard to the management of any public commercial company, would quickly run down the value of the shares, and we are hardly surprised to find that at the meeting in question the Treasurer had to announce a deficit on the general account of £1,186, which, added to that of £384 brought down from last year, made a total adverse balance of £1,570.

